# 

**Published:** 1846-02

**Authors:** 


CONTENTS:

Mortality of Chicago, by Wm. B. Herrick, Professor of Anatomy, - - - - 161

Bibliographical Notices.
The monthly Miscellany and Journal of health, by W. M. Cornell, M. D., - - 163

Medical Intelligence.
Medical Schools, - - 164
A New Medical Society, - 165
Our Journal—Its Enlargement, 165

Practical Medicine, &c.
Thymic Asthma, by M. Trousseau, - - - - 165
The Use of Phosphate of Ammonia in Gout and Rheumatism, by T. H. Buckler, M. D., 168
On the Therapeutic Action of Arsenic, by M. Boudin, - 171
Sunlight and Health, - - 173
Epidemic Small Pox, - 174
Fictitious Catalogues of Medical Students, - - - - - 175
The employment of Blisters in acute diseases of the Brain, by Dr. Tritshler, - - 176
Lithontriptic Action of the Uva Ursi, by Dr. Fenolio, - 176
A new Hæmostatic Means—Sheep Brains, - - - - - 176

